# Canine paroxysmal dyskinesia: distribution of etiological subtypes and structural and reactive causes in a retrospective tertiary care cohort

**DOI:** 10.3389/fvets.2026.1846296

**Published:** 2026-07-31

**Authors:** Anna Dellwig, Nina Meyerhoff, Casey B. Rogers, Holger A. Volk, Andrea Tipold, Jasmin N. Nessler

**Affiliations:** 1 Department of Small Animal Medicine and Surgery, University of Veterinary Medicine Hannover, Hannover, Germany; 2 Veterinary Clinic for Small Animals, Norderstedt, Germany

**Keywords:** basal ganglia, canine movement disorder, etiological classification, hypocalcemia, reactive paroxysmal dyskinesia, structural paroxysmal dyskinesia

## Abstract

**Introduction:**

Canine paroxysmal dyskinesia is an episodic movement disorder characterized by transient dystonia, dyskinesia and tremor in dogs with preserved consciousness, yet its differentiation from focal epilepsy remains challenging and a structured diagnostic framework comparable to that used in epilepsy is lacking.

**Methods:**

This retrospective, single-center study evaluated the distribution of etiologies in dogs presenting with paroxysmal dyskinesia to a tertiary referral center between 2012 and 2023. Medical records were systematically screened, and dogs were included if episodic abnormal movements fulfilled established clinical criteria while cases with impaired consciousness, postictal signs or insufficient documentation were excluded. Diagnostic data comprised history, neurological examination, laboratory testing, serology for gluten sensitivity, advanced imaging and cerebrospinal fluid analysis when available, and cases were categorized into suspected idiopathic, genetic (suspected and confirmed), gluten-sensitive, reactive or structural groups.

**Results:**

Seventy dogs met inclusion criteria. Suspected idiopathic forms predominated (61.4%), followed by genetic (suspected and confirmed; 22.9%) and gluten-sensitive (12.9%) forms, whereas reactive and structural causes were rare (1.4% each). Across groups, dystonia with concurrent tremor and episodic gait or postural abnormalities represented the dominant clinical topographical distribution. Neither age at onset nor topographical distribution of signs reliably differentiated etiologies. Body weight differed significantly between groups.

**Discussion:**

These findings demonstrate that most cases represent diagnoses of exclusion and that clinical presentation alone is insufficient for etiological classification, underscoring the need for a standardized, tiered diagnostic strategy integrating basic laboratory evaluation, targeted genetic testing in predisposed breeds and early assessment for gluten sensitivity, with advanced diagnostics reserved for selected cases.

## 1 Introduction

Canine paroxysmal dyskinesia (cPD) is a group of episodic, self-limiting movement disorders in dogs (1). Within these episodes, affected dogs exhibit dyskinesia, dystonic movements, and focal or generalized tremors while maintaining normal consciousness (1). A postictal phase is absent, and autonomic signs are generally not observed apart from occasional gastrointestinal manifestations (1–3). Episodes may occur at rest (paroxysmal nonkinesigenic dyskinesia), after sudden movements (paroxysmal kinesigenic dyskinesia), or after strenuous physical activity (paroxysmal exercise induced dyskinesia) (4). Reported etiologies of cPD include genetic and presumed genetic forms, dietary-associated variants, reactive and structural disorders, as well as unknown causes (1, 3–25).

Despite these defining characteristics, clinical differentiation from epilepsy, particularly focal seizures, remains challenging in everyday practice and misclassification carries the risk of inappropriate therapeutic decisions (26, 27). However, at present, no structured framework exists to guide the systematic diagnostic evaluation of dogs presenting with cPD, as is available for epilepsy (28). The development of such an approach requires knowledge of the relative frequency of the different etiologies within a clinical population. Only by understanding which underlying conditions predominate and which occur rarely can diagnostic priorities be defined in an evidence-based manner.

The aim of the present study was therefore to identify additional structural and reactive causes and to investigate the distribution of etiologies in dogs presenting with cPD at a tertiary referral center.

## 2 Materials and methods

### 2.1 Selection of animals

This is a retrospective, single-centre study using the digital medical records of the *Clinic for Small Animals of the University of Veterinary Medicine Hannover.* The electronic record system was searched for dogs presented between 2012 and 2023 using the search terms “paroxys*”, “dyskin*”, “movement disorder”, “scottie cramp”, “seizures”, “ataxia”, “CNS disease” and “inflammatory CNS diseases”. Cats were excluded. All identified medical records were subsequently reviewed individually and selected according to the predefined inclusion and exclusion criteria.

Dogs were eligible for inclusion if they exhibited episodic abnormal movements consistent with cPD, defined as episodic dyskinesia, dystonic movements, with or without focal or generalized tremors, according to previously established criteria (1).

Exclusion criteria comprised reduced consciousness during episodes, autonomic signs such as urination, defecation, or salivation not associated with vomiting, postictal signs, or missing essential information required to classify paroxysmal dyskinesia (status of consciousness, autonomic signs, pre-/postictal changes). Dogs presenting mydriasis as the sole autonomic sign were not excluded, given that mydriasis may reflect a stress-related sympathetic response to cPD-associated clinical signs, as previously described in cats (29). Dogs that exhibited salivation prior to vomiting during episodes were not excluded as gastrointestinal signs are commonly reported in cPD (2, 30, 31).

For each case, the following data were collected: medical history, results of general and neurological examination, blood analysis (complete blood count, clinical chemistry, and electrolytes, and if available bile acids, ammonia, and thyroid hormone levels), serological testing for anti-gluten antibodies, magnetic resonance imaging (MRI), computed tomography (CT), and cerebrospinal fluid (CSF) analysis. As a minimum diagnostic requirement for study inclusion, a general physical examination, neurological examination, and at least hematological examination had to be available. Assessment of possible toxic exposure was based on the clinical history provided by the owners and the overall clinical presentation, as standardized diagnostic tests for many intoxications are not routinely available in veterinary medicine.

All dogs were categorized into one of six etiological subgroups: suspected idiopathic, reactive, structural, confirmed genetic, suspected genetic, or gluten sensitive (Table 1). As a result, the different categories were characterized, and their similarities and differences were systematically identified.

**Table 1:** Overview of the classification criteria applied for the etiology of canine paroxysmal dyskinesia (cPD).

Criteria	Classification
Suspected idiopathic	Unremarkable interictal examination, unremarkable blood examination (CBC, clinical chemistry, bile acids or ammonia, electrolytes, thyroid hormones), physiological titers of anti-gluten-antibodies or no improvement on gluten-free diet, and unremarkable MRI brain and CSF examination. Minimum of diagnostic workup: unremarkable interictal examination, and CBC
Confirmed genetic	Positive breed specific genetic test
Suspected genetic	Breed predisposition, but no commercially available genetic test, or available test not performed
Gluten sensitive	Serological evidence of gliadin-IgG and transglutaminase-2-IgA with a ratio of >0.8 (positive) with known or unknown response to a gluten-free diet; serological results with a ratio of 0.6–0.8 (questionable: either transglutaminase-2 IgA or gliadin IgG within this range) and clinical improvement following a gluten-free diet; serological testing not performed, but clinical improvement following a gluten-free diet
Structural	Structural changes in MRI/CT of the brain or abnormal CSF results
Reactive	Systemic metabolic disorders (clinically relevant changes in electrolyte, triglycerides or blood-glucose-levels, elevated pre- or postprandial bile acids and/or ammonia, hypothyroidism, hyper- or hypocortisolism or other endocrinologic disease), intoxications, drug induced

### 2.2 Statistical analysis

Descriptive statistics were calculated for clinical signs of cPD, body weight, and age at onset of clinical signs. The assumption of normality for continuous variables (age, weight) was assessed visually using Q–Q plots. As the data were not normally distributed, non-parametric methods were applied.

For statistical analysis, etiologies were grouped as follows: the first group included both confirmed and suspected genetic cases; the second included suspected idiopathic cases; and the third group was gluten sensitive. Cases with reactive or structural etiologies were excluded from statistical analysis due to low sample size (*n* = 1 per group).

Clinical signs of cPD were analyzed based on the involvement (yes/no) of the trunk, head, and limbs, as well as the presence (yes/no) of ataxia, tremor, and dystonia. Differences in age and body weight between the three classification groups were assessed using the Kruskal–Wallis test. Associations between classification groups and categorical clinical signs were analyzed using Chi-square tests. A two-sided *p*-value <0.05 was considered statistically significant. All analyses were performed using SAS software, version 9.04.

## 3 Results

**Figure 1:**
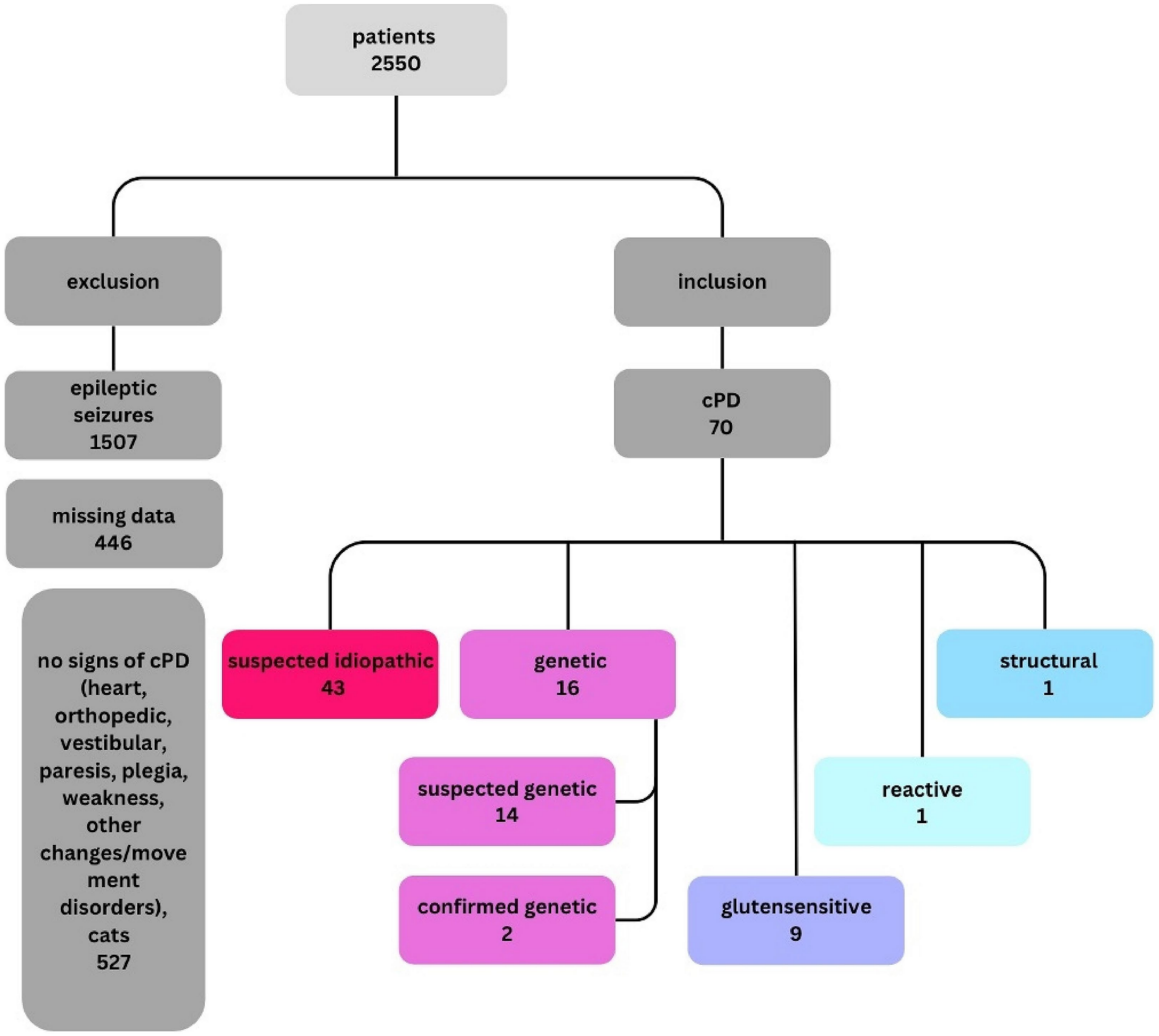
Flowchart of patient selection and classification. From the initial 2,550 patients, 1,507 dogs with focal or generalized seizures were excluded, 446 dogs were excluded due to missing data, 527 patients were excluded because they did not show signs of canine paroxysmal dyskinesia (cPD), had other diseases instead, or other movement disorders (e.g., head bobbing, ataxia, tremor, myoclonus, fly biting, head shaking, transient decerebrate posture, excessive air licking, and obsessive pacing). Among these 527 patients also 26 cats were excluded. In total 70 dogs with cPD were included.

The search revealed 2,550 patients; 26 cats were excluded. Five hundred one dogs were excluded because they showed no signs of cPD. One thousand five hundred seven were excluded because they showed signs of epileptic seizures (generalized tonic–clonic seizures, orofacial seizures, changes in consciousness, autonomic signs, and/or postictal changes). Four hundred forty-six were excluded due to insufficient data to allow classification of the episodes (Figure 1).

A total of 70 dogs were included in the study (Figure 1). Among the included patients were 31 male (13/31 neutered) and 39 female (18/39 neutered). There were 36 different breeds [mixed breed (*n* = 12); Labrador Retriever (*n* = 8); Bolonka Zwetna (*n* = 5); Poodle, Malinois (*n* = 3 each); Bichon Frise, Beagle, Cavalier King Charles Spaniel, Pomeranian, Chihuahua, Dachshund, Yorkshire Terrier an Jack Russel Terrier (*n* = 2 each); French Bulldog, Norwich Terrier, Shetland Sheepdog, Husky, Patterdale Terrier, Scooter, Small Munsterlander, Soft Coated Wheaten Terrier, Terrier, Nova Scotia Duck Tolling Retriever, Cocker Spaniel, Podenco, Lhasa Apso, Goldendoodle, Magyar Viszla, Shar Pei, Border Terrier, ShihTzu, Elo, Havanese, Pug, Bavarian Mountain Hound and Scent Hound (*n* = 1 each)]. The age was between 0 and 9 years.

### 3.1 Suspected idiopathic paroxysmal dyskinesia

Of 70 dogs, 43/70 (61.4%) were classified as suspected idiopathic cPD. The cohort comprised 29 females (13/29 neutered) and 14 males (7/14 neutered). Mixed breed dogs were most common (10/43), followed by Bolonka Zwetna (5/43). All other breeds were represented by ≤2 individuals.

**Figure 2:**
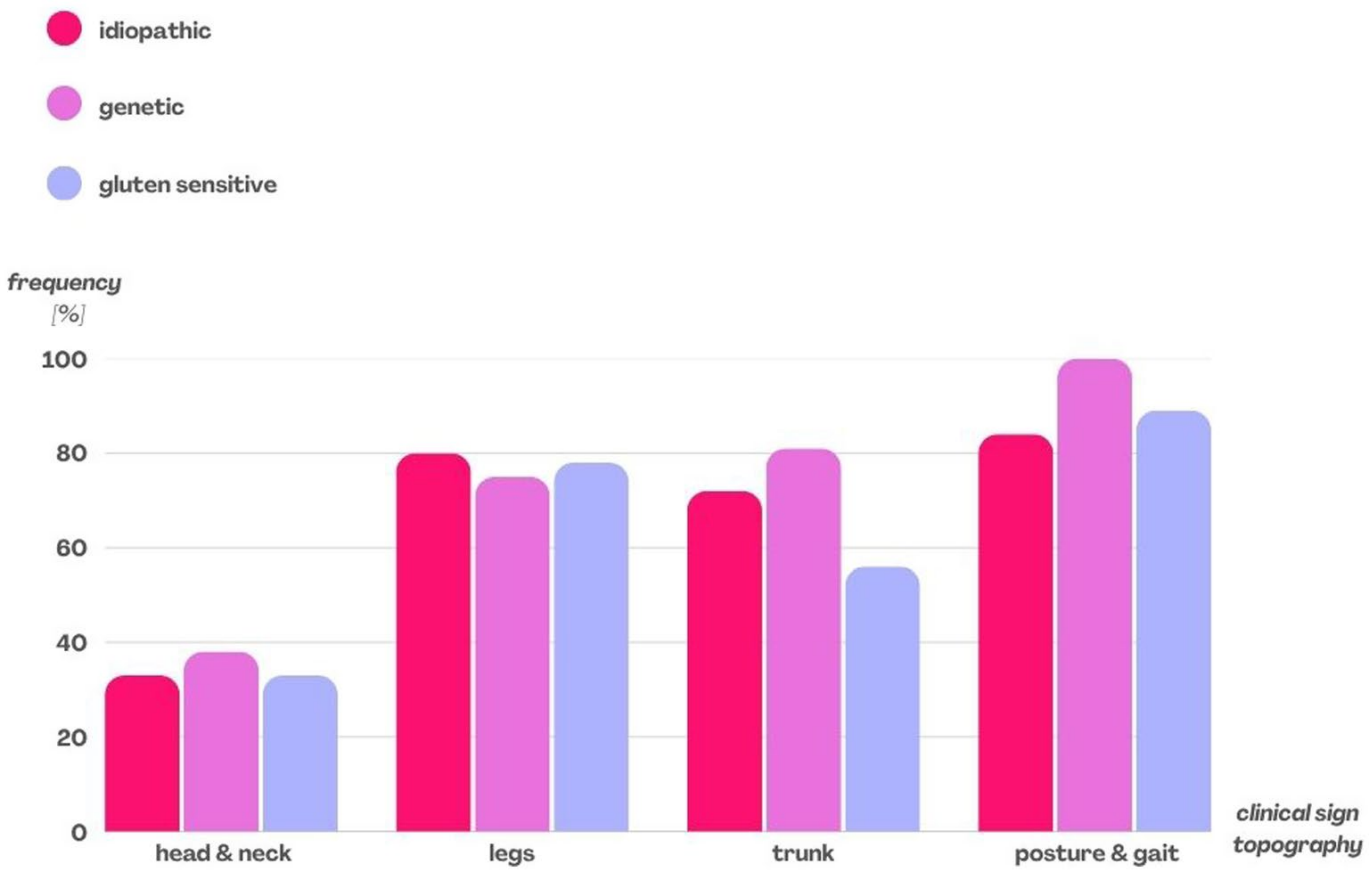
Topographical distribution of clinical signs in dogs with suspected idiopathic (*n* = 43), genetic (suspected and confirmed; *n* = 16), and gluten-sensitive (*n* = 9) canine paroxysmal dyskinesia. The *x*-axis displays the clinical sign topography (position/gait abnormalities, limb involvement, axial body involvement, head and neck localization), and the *y*-axis indicates the frequency in percent (%). In the idiopathic group, position/gait abnormalities were observed in 83.7%, limb involvement in 79.1%, axial involvement in 72.1%, and head and neck localization in 32.6% of dogs. In the genetic group (suspected and confirmed), position abnormalities occurred in 100%, axial involvement in 81.3%, limb involvement in 75%, and head and neck localization in 37.5%. In the gluten-sensitive group, position abnormalities were present in 88.9%, limb involvement in 77.8%, involvement of the trunk in 55.6%, and head and neck localization in 33.3% of dogs.

Median age at onset was 3 years (range 0–9 years; age unknown in 4 dogs). Episodic abnormalities most frequently involved posture and/or gait (36/43; 83.7%), followed by limb involvement (34/43; 79.1%) and trunk involvement (31/43; 72.1%). Head and neck signs were reported in 14/43 dogs (32.6%) (Figure 2). Limb manifestations were predominantly dystonic (15/43), while trunk involvement mostly included kyphosis (11/43), tremor (8/43), or stiffness (6/43). Head and neck signs were most frequently characterized by tremor (6/43). Episodic abnormalities in posture and/or gait were most reported as recumbency (*n* = 7/43) and ataxia (*n* = 6/43). Autonomic signs were uncommon and included vomiting (3/43), mydriasis (3/43), and salivation before vomiting (1/43). Routine hematology (performed in 43/43 dogs) and biochemistry (performed in 43/43 dogs), CSF analysis (performed in 11/43 dogs), gluten antibody testing (performed in 12/43 dogs), and advanced imaging MRI (performed in 11/43 dogs)/CT (performed in 0/43 dogs) did not reveal clinically relevant abnormalities. Minimal MRI changes were described in 6/11 dogs, including one breed-specific malformation (incomplete fontanelle closure), none considered clinically relevant. Three dogs showed elevated or questionably elevated gliadin-IgG and/or transglutaminase-2-IgA concentrations; however, no clinical improvement was observed under a strict gluten-free diet.

### 3.2 Genetic paroxysmal dyskinesia (suspected and confirmed)

Sixteen out of seventy (22.9%) dogs were classified as having genetic cPD (14/16 suspected genetic cPD). Abnormalities of the head and neck were reported in 6/16 dogs (37.5%), limb involvement in 12/16 (75%), trunk involvement in 13/16 (81.3%), and posture and gait abnormalities in 16/16 dogs (100%) (Figure 2).

Two Cavalier King Charles Spaniel (1/2 female) were classified as confirmed genetic cPD based on detection of the BCAN microdeletion associated with the previously described “episodic falling” defect. Age at onset was 5 years in one dog and unknown in the other. One dog exhibited episodic increased jaw tone, tonic–clonic movements of the left pelvic limb, and generalized body tremor occurring during lateral recumbency, whereas the second dog presented with episodic incoordination and sudden falls. Interictal neurological examination was unremarkable in one dog, whereas the other showed cervical pain. Hematology and clinical chemistry revealed no clinically relevant abnormalities. MRI (performed in one dog) demonstrated mild Chiari-like malformation and syringohydromyelia, considered incidental. CSF analysis was not performed in either dog.

Fourteen out of sixteen were classified as suspected genetic cPD including 8 Labrador Retrievers [2/8 female (1/2 neutered), 6/8 male (3/6 neutered)], one male Border Terrier, two neutered male Jack Russell Terriers, one male Norwich Terrier, one female Shetland Sheepdog, and one neutered female Soft Coated Wheaten Terrier. Median age at first onset ranged from 1 to 6 years.

Within the Labrador Retrievers, episodic head and neck involvement was reported in 3/8 dogs (37.5%), including tremor (*n* = 1/8), head sway (*n* = 1/8), and combined tremor with head sway (*n* = 1/8); no further specification was available in 5/8 dogs (Figure 2, Table 2). Clinical signs involving one or more limbs were described in all dogs (8/8; 100%). Dystonia was most frequent (*n* = 2/8), while additional manifestations (each *n* = 1/8) included dystonia with chorea, dystonia with paddling, limb buckling with stiffness, hind limb weakness, isolated stiffness, and tremor with “flicking” (Figure 2, Table 2). Truncal involvement occurred in all dogs (8/8 dogs; 100%), most commonly tremor (*n* = 5/8), followed by kyphosis (*n* = 2/8) and stiffness (*n* = 1/8) (Figure 2, Table 2). Abnormalities of posture and/or gait were reported in all dogs (8/8; 100%) and included sudden falls or lying down (*n* = 4/8), ataxia (*n* = 3/8), and maintenance of a standing posture during episodes (*n* = 1/8) (Figure 2, Table 2). Clinical presentation among the remaining suspected genetic breeds were as followed. The Border Terrier showed episodic head and truncal sway accompanied by ataxia; limb involvement was not documented. In the two Jack Russell Terrier, one dog exhibited episodic truncal tremor and sway, whereas the second presented with episodic prayer posture; head, neck, and limb involvement were not specified. The Norwich Terrier demonstrated episodic head sway, truncal sway, and stiffness of the thoracic limbs. The Shetland Sheepdog presented with episodic limb dystonia, truncal stiffness, and collapse; posture and gait were characterized by episodic ataxia, while head and neck involvement was not reported. The Soft Coated Wheaten Terrier exhibited episodic limb dystonia and generalized stiffness; posture during episodes was described as maintained standing, and head/neck signs were not documented. Autonomic signs during episodes were observed in 3/14 dogs (21.4%) and consisted of vomiting; one of these dogs additionally exhibited mydriasis. Interictal neurological examination was available for 13/14 dogs and was unremarkable in all evaluated cases. Hematological (performed in 14/14 dogs) and clinical chemistry (performed in 14/14 dogs), MRI (performed in 6/14 dogs) and CSF analysis (performed in 5/14 dogs) did not demonstrate clinically relevant abnormalities. Gluten antibody testing (performed in 2/14 dogs) showed elevated titers in one dog and mildly elevated titers in the other dog. Both dogs showed no clinical improvement following implementation of a gluten free diet.

**Table 2:** Topographic distribution of dominant episodic clinical signs.

Topography	Suspected idiopathic	Genetic (suspected and confirmed)	Gluten sensitive
Head/neck	Tremor	Head sway	Tremor
Limbs	Dystonia	Dystonia	Dystonia
Trunk	Kyphosis	Tremor	Tremor
Posture/gait	Recumbency	Sudden falls/recumbency	Recumbency

### 3.3 Gluten-sensitive paroxysmal dyskinesia

Nine of 70 dogs (12.9%) were classified as gluten-sensitive cPD. The cohort comprised 4/9 females (1 neutered) and 5/9 males (1 neutered). Breeds included two Poodle and one each of Nova Scotia Duck Tolling Retriever, Shih Tzu, Labrador Retriever, Pomeranian, Belgian Malinois, Bichon Frisé, and one mixed-breed dog. Median age at onset was 3 years (range 0–5 years). Episodic head and neck involvement was reported in 3/9 dogs (33.3%) and consisted of tremor (*n* = 2/9) and retrocollis (*n* = 1/9) (Figure 2, Table 2). Clinical signs involving one or more limbs occurred in 7/9 dogs (77.8%), most commonly dystonia (*n* = 3/9). Additional individual manifestations (each n = 1/9) included dystonia with athetosis, dystonia with chorea, ballism, and twitching (Figure 2, Table 2). Truncal involvement was described in 5/9 dogs (55.6%) and included combined kyphosis with tremor, kyphosis with scoliosis, tremor with stiffness, isolated stiffness, and isolated tremor (each *n* = 1/9) (Figure 2, Table 2). Posture and/or gait abnormalities were reported in 8/9 dogs (88.9%), including recumbency (*n* = 4/9), ataxia (*n* = 2/9), standing (*n* = 1/9), and staggering (*n* = 1/9) (Figure 2, Table 2). Interictal neurological examination was unremarkable in 6/9 dogs. Individual abnormalities included hyperalgesia at L3/L4 (*n* = 1/9) and altered pupillary reflexes with hippus (*n* = 1/9); no data were available in one dog. Serological testing supported gluten sensitivity in 7/8 tested dogs; one additional dog showed mildly elevated titers but clinical response to dietary modification. Overall, clinical improvement under a gluten-free diet was documented in 6/9 dogs. Hematology (performed in 9/9 dogs), clinical chemistry (performed in 9/9 dogs), MRI (performed in 3/9 dogs), and CSF analysis (performed in 3/9 dogs) revealed no clinically relevant abnormalities (*n* = 9/9).

### 3.4 Structural paroxysmal dyskinesia

One of 70 dogs (1.4%) was classified as structural cPD (MRI was performed in 22/70 dogs). The affected dog was an 8 week old intact male Small Munsterlander, representing the youngest age at onset within the cohort. Episodic clinical signs involved multiple body regions and included head and neck tremor, limb dystonia, recumbency, truncal sway, and mild opisthotonus. Interictal neurological examination was unremarkable. Advanced diagnostics revealed structural and inflammatory abnormalities. MRI demonstrated pronounced deep sulci, particularly within the temporal and frontal lobes, suggestive of degenerative or atrophic changes. CSF analysis showed mixed cell pleocytosis. PCR testing for infectious agents (Neospora, Toxoplasma, canine distemper virus) was negative. Hematology and clinical chemistry revealed no clinically relevant abnormalities. Anti-gluten antibody testing was not performed. Based on imaging and CSF findings, suspected diagnoses included meningoencephalitis of unknown origin or an undefined inherited neurodegenerative disorder. Long-term clinical outcome was not available due to lack of follow-up.

### 3.5 Reactive paroxysmal dyskinesia

One of 70 dogs (1.4%) was classified as reactive cPD. The affected dog was a female mixed breed with onset of clinical signs at 3 years of age. Episodic manifestations included head and neck tremor, limb dystonia, and ataxia; truncal involvement was not documented. Interictal neurological examination was unremarkable. Biochemical analysis revealed marked hypocalcemia (0.8 mmol/L; reference interval 1.2–1.6 mmoL/L). Hypoparathyroidism was diagnosed based on parathyroid hormone concentrations at the lower end of the reference range, and calcium supplementation was initiated. Gluten sensitivity testing yielded equivocal results, and a gluten-free diet was additionally introduced. Following treatment, clinical signs improved and serum calcium normalized. After subsequent gastrointestinal illness, one additional episode occurred in association with a calcium concentration of 1.15 mmoL/L. MRI and CSF analysis were not performed.

### 3.6 Comparison of topographical distribution and demographic characteristics between groups

Groups did not significantly differ in age of onset, trunk, head, or limb involvement, nor in the presence of ataxia, tremor, or dystonia, or in age at onset of clinical signs (all *p* > 0.05). However, groups differed significantly in body weight (*p* = 0.0261; see Table 3). The median body weight was 22 kg in dogs with genetic cPD, 17 kg in dogs with gluten-sensitive cPD, and 10 kg in dogs with suspected idiopathic cPD.

**Table 3:** Descriptive statistics and comparison of continuous variables between classification groups.

Characteristic	Suspected idiopathic	Genetic (suspected and confirmed)	Gluten sensitive	*p*
Age of onset of clinical signs [years] median (ICQ), *n*	3 (2–4) *n* = 39	4 (3–5) *n* = 14	3 (1–3) *n* = 9	0.1587
Body weight [kg] median (IQR), *n*	10 (4.6–15.6) *n* = 43	22 (8.8–29.6) *n* = 15	17 (7.6–23.4) *n* = 9	0.0261

## 4 Discussion

This retrospective study provides a structured overview of canine paroxysmal dyskinesia (cPD) based on the classification of 70 affected dogs according to diagnostic work-up and presumed etiology. The application of a classification system adapted from epilepsy classifications aims to systematically consider both epileptic disorders and cPD within the diagnostic work-up, consistently including cPD as a differential diagnosis for reducing misclassification.

Within the cohort, suspected idiopathic forms constituted the largest group, followed by genetic and gluten-sensitive variants, whereas structural and reactive causes were identified only rarely.

Differentiation between suspected idiopathic, genetic (suspected and confirmed), and gluten sensitive cPD proved to be particularly challenging. No relevant differences were observed between the classification groups with regard to age at disease onset or the topographical distribution of movement disorders. Dystonia represented the dominant clinical hallmark across all groups and frequently occurred in combination with tremor as well as episodic gait or postural abnormalities. The expression and combination of clinical signs varied individually, without consistent etiology-specific patterns, highlighting the need for a systematic diagnostic approach. A significant difference in body weight was observed between the classification groups. Dogs with genetic cPD had the highest median body weight, followed by dogs with gluten sensitive cPD. Dogs with suspected idiopathic cPD had the lowest body weight. The genetic group consisted predominantly of Labrador Retrievers (suspected genetic), a breed characterized by a comparatively larger body mass. This raises the question of whether body weight serves as an indirect indicator of breed distribution or whether the observed association truly reflects an effect of body weight itself. To date, gluten sensitive cPD has only been described in association with a specific breed in Border Terriers, which have a substantially lower average body weight than the dogs represented in the gluten-sensitive cPD cohort of the present study (31). In previous studies including mixed cohorts, the median body weight of serological positive dogs was 16.06 kg, which may further support this hypothesis (20). The increased proportion of smaller dogs within the suspected idiopathic category additionally suggests that diagnostic markers may be lacking particularly in smaller breeds, highlighting the need to more specifically address this population in future investigations.

The high proportion of suspected idiopathic cases reflects both the presence of disorders with currently unknown pathogenesis and limitations of diagnostic evaluation inherent to the retrospective study design. Dogs presenting with autonomic signs were excluded to reduce the risk of misclassifying epileptic seizures as cPD (1). Among the excluded cases, there may have been dogs with gluten-sensitive cPD in which salivation can occur during episodes. To distinguish gastrointestinal signs from isolated autonomic manifestations, salivation was only accepted when reported in association with vomiting. As a result, the prevalence of this subtype may have been underestimated (14, 20).

Considering the present findings and the observed distribution of etiologies, implementation of a structured diagnostic approach in dogs with cPD appears warranted.

Although suspected idiopathic cPD represents the most prevalent category, it should be regarded as a diagnosis of exclusion. The diagnostic work-up should begin with a thorough neurological examination to identify interictal abnormalities that may indicate an underlying structural disorder of the central nervous system. In the presence of known breed-specific predispositions or previously described mutations, targeted genetic testing should be integrated early into the diagnostic work-up (6, 12, 17, 19, 32–34). Implementation of a strictly gluten-free diet represents an additional diagnostic and therapeutic measure that should be considered early in the clinical work up. Serological testing may support the diagnosis and can be used for follow up assessment (20). Persistently elevated antibody titers should prompt verification of strict dietary adherence before reconsidering the etiological classification. Structural and reactive forms of cPD play a subordinate role, but should be considered in the differential diagnostic process, particularly in cases with abnormalities between episodes, progressive disease courses, or concurrent additional clinical signs of an underlying disorder. Therefore, hematological and serum biochemical analyses are recommended. Isolated case reports exist, including paroxysmal dyskinesias in cats associated with hyperthyroidism and in dogs with hypocalcemia, which may present with both epileptic seizures and atypical, seizure-like episodes without loss of consciousness or autonomic signs (35, 36). Advanced diagnostic procedures, including MRI and CSF, should be considered in cases with abnormal neurological findings (28). Due to the retrospective nature of the study, structural cPD may be underrepresented, as in dogs with prominent structural neurological diseases, such as structural epilepsy, cPD may not be recognized or reported by owners and may therefore be overlooked.

Several limitations need to be considered when interpreting the proposed subgroup classifications. Due to the current lack of universally accepted diagnostic criteria and reliable confirmatory biomarkers for canine paroxysmal dyskinesia, classification of cases was primarily based on clinical presentation, previously described phenotypic characteristics, and classifications proposed in the existing literature. Consequently, the proposed etiological groupings should be interpreted as presumptive rather than definitive classifications.

The categorization of dogs into the presumed genetic group was mainly based on breed predisposition and previously reported phenotypes. However, the absence of confirmed causative genetic variants in most breeds currently limits the diagnostic certainty of this classification. Future studies identifying and validating potential genetic variants in predisposed breeds may help to clarify the true proportion of affected dogs carrying disease-associated mutations.

Furthermore, the interpretation of gluten-sensitive cPD remains limited. In human medicine, elevated transglutaminase-2 IgA concentrations have also been reported in other immune-mediated disorders independent of gluten sensitivity, which may limit the specificity of serological testing in dogs as well (37–39). Additionally, not all dogs underwent standardized dietary trials or gluten-sensitivity testing, further limiting interpretation of this subgroup, as changes in clinical signs were based on subjective owner assessments. An additional limitation is that some dogs with positive gluten-associated serology lacked sufficient follow-up information regarding their clinical response to a strict gluten-free diet. Consequently, some of these cases may potentially have been more appropriately classified within the suspected idiopathic cohort if no clinical improvement had occurred, which may have influenced the distribution of cases between the proposed subgroups. These findings highlight the need for prospective studies using standardized diagnostic protocols and more reliable diagnostic biomarkers.

An additional limitation of the present study is the inconsistent availability of long-term follow-up data. Due to the retrospective nature of the study, follow-up examinations and reassessments were not standardized and were unavailable for all dogs. Consequently, the possibility that alternative or progressive neurological diseases may have emerged later in some cases cannot be completely excluded. This limitation should be considered when interpreting the proposed classifications and diagnostic assumptions.

Overall, the present study is based on the diagnostic tools and classifications currently available and should therefore be interpreted as exploratory in nature. With the future identification of additional biomarkers, genetic variants, and improved diagnostic approaches, particularly within prospective study designs, the distribution and classification of cases may change. This should be considered when interpreting and evaluating the findings of the present study.

The study underscores the need for prospective investigations using standardized diagnostic protocols to improve comparability between studies and to more precisely characterize etiological associations. In particular, the high number of suspected idiopathic cases suggests that currently unidentified factors may play a relevant role, the elucidation of which will require future molecular genetic and functional studies. Furthermore, body weight, body size, and breed should be systematically analyzed in future research, accounting for potential interactions, to determine whether body weight exerts an independent biological influence on etiology or merely serves as an indirect marker of breed-specific predispositions. The establishment of a uniform diagnostic guideline could further refine the classification of cPD and thereby enhance both scientific understanding and clinical management of this heterogeneous disorder.

## 5 Conclusion

Suspected idiopathic cPD was most frequently represented in the present cohort, followed by genetic (suspected and confirmed) and gluten-sensitive forms, whereas structural and reactive causes were rare. Suspected idiopathic cPD should be regarded as a diagnosis of exclusion. Age of onset and clinical presentation did not allow reliable differentiation between etiological subgroups, underscoring the relevance of a structured diagnostic approach. A stepwise diagnostic work-up including neurological examination, basic laboratory testing, targeted genetic analysis, and evaluation for gluten sensitivity, appears appropriate, with advanced diagnostics reserved for selected cases.

## Data Availability

The original contributions presented in the study are included in the article/supplementary material, further inquiries can be directed to the corresponding author.
